# Bone Marrow Mesenchymal Stem Cells Ameliorates Seawater-Exposure-Induced Acute Lung Injury by Inhibiting Autophagy in Lung Tissue

**DOI:** 10.1155/2014/104962

**Published:** 2014-08-19

**Authors:** Qiu-ping Liu, Dang-xia Zhou, Li Sun, Luo Ling, Chang-gui Wu, Pu Lin, Shui-ping Han

**Affiliations:** ^1^Third Ward of VIP, 323 Hospital of PLA, Xi'an 710054, China; ^2^Pathology Department, Medical School, Xi'an Jiaotong University, Xi'an 710061, China; ^3^Ward of VIP, Shaanxi People's Hospital, Xi'an 710068, China; ^4^Department of Endocrinology, The First Hospital of PLA, Lanzhou 730000, China; ^5^Department of Respiratory Medicine, Xijing Hospital, Fourth Military Medical University, Xi'an 710038, China; ^6^Electric Power Science Research Institute of Shaanxi Province, Xi'an 710054, China

## Abstract

Seawater drowning can lead to acute lung injury (ALI). Several studies have shown that bone marrow mesenchymal stem cells (BMSC) treatment could attenuate ALI. However, the mechanisms underlying this phenomenon still remain elusive. Therefore, this study aimed to investigate whether BMSC treatment can ameliorate seawater-induced ALI and its underlying mechanisms in a rat model. In this study, arterial blood gas, lung weight coefficient, and TNF-*α*, and IL-8 in bronchoalveolar lavage fluid (BALF), as well as histopathology examination, were used to detect the lung injury of seawater exposure. Moreover, western blot and RT-PCR were used to explore autophagy in lung tissues. The results demonstrated that seawater exposure induced ALI including impaired arterial blood gas, pulmonary edema, histopathologic changes, and inflammatory response in lung tissues. What is more, these changes were partly ameliorated by BMSC treatment through inhibition of autophagy in lung tissues. The application of BMSC may be a potential effective treatment for seawater-induced ALI.

## 1. Introduction

Drowning is one of important causes of accidental deaths. It has been shown that drowning accounts for approximately half a million deaths each year in the world [1]. Acute pulmonary injury (ALI)/and its more severe form, acute respiratory distress syndrome (ARDS), are common symptoms of drowning [2]. As seawater has high osmolarity and its NaCl concentration is about 3-fold of that in physiologic saline, ALI, including lung edema, hypoxemia, and inflammatory reaction, is more serious in seawater drowning compared to those in freshwater drowning [3]. However, currently there are no effective strategies to treat it.

Bone marrow mesenchymal cells (BMSCs) are multiple differentiation progenitor cells derived from bone marrow, which have the ability to differentiate into endothelial cells, vascular smooth muscle cells, bone, cartilage, muscle, and other connective tissues [4]. BMSCs are being studied with increasing intensity because of their potential use as a therapeutic tool [5]. Several studies have demonstrated that the BMSC transplantation might attenuate ALI/ARDS in lung tissue by decreasing mortality rate and maintaining the normal pulmonary endothelial and epithelial function as well as participating in modulating the inflammatory responses that are involved in ALI [6–9]. BMSC-based therapy seems to be a promising treatment for ALI/ARDS. However, little is known about the therapeutic effects of BMSC transplantation on ALI induced by seawater exposure.

Although BMSCs are being studied as potential therapeutic tool for ALI, the discrete mechanisms underlying this phenomenon still remain unclear. Autophagy is an evolutionarily conserved physiological process which provides a membrane-dependent mechanism for the sequestration, transport, and lysosomal turnover of subcellular components, including proteins and organelles [10]. Autophagy occurs at low basal levels in cells to execute homeostatic function. However, it could be rapidly upregulated under various stress conditions such as hypoxia, ischemia, oxidative stress, and endoplasmic reticulum stress [11]. Recently, it is increasingly clear that autophagy is relevant to many pulmonary diseases including ALI, and autophagy-blocking agents might rescue cell death and ameliorate ALI [12–14]. Our earlier studies also demonstrated that seawater exposure triggered autophagy, and autophagy might be a damaging factor responsible for ALI induced by seawater [15]. However, as far as is known, there is little evidence of whether BMSC transplantation has therapeutic effects on seawater-induced ALI by inhibiting autophagy in lung tissues.

Therefore, the current study was aimed to investigate whether BMSC treatment can ameliorate seawater-induced ALI and its underlying mechanisms, focusing on the changes of autophagy in particular. Understanding the role of BMSC in the seawater exposure will contribute to further disclosing the therapy effects of BMSC in ALI/ARDS.

## 2. Materials and Methods

### 2.1. Animals Preparation

Healthy adult male Sprague-Dawley rats weighing 180–200 g from Experimental Animal Center of Xi'an Jiaotong University were obtained and housed in solid-bottomed polycarbonate cages in SPF animal laboratory with a temperature 21–25°C and a relative humidity of 40–60%. Rats were acclimatized at a 12 h light/12 h dark cycle and fed a standard diet and tap water* ad libitum* before the experiments. Experiments were performed in accordance with the Animal Experimentation Committee Regulation of Xi'an Jiaotong University.

### 2.2. Seawater and Bone Marrow Stem Cells (BMSCs) Preparation

According to the methods of previous study [16], seawater (osmolality 1300 mmol/l, SW 1.05, pH 8.2, NaCl 26.518 g/l, MgSO_4_ 3.305 g/l, MgCl_2_ 2.447 g/l, CaCl_2_ 1.141 g/l, KCl 0.725 g/l, NaHCO_3_ 0.202 g/l, and NaBr 0.083 g/l) was prepared.

BMSCs (RASMX-01001) of SD rats were purchased from Cyagen Biotechnology Ltd. in Guangzhou, China. The cells were further cultured in Iscove's modified Dulbecco's medium (IMDM, Hyclone) with 20% fetal bovine serum at 37°C. After subculturing 3–5 times, the BMSCs were used for the following experiments.

### 2.3. Establishment of Seawater-Induced Acute Lung Injury Model

Seawater group (SG): According to the methods of Han et al. study [16]. The rats in the experimental groups were anesthetized with 20% urethane (5 mL/kg) intraperitoneally and maintained in the supine position during experiments. A tube was inserted into trachea through a tracheostomy and then 3 mL/kg of seawater was aspirated into trachea within 5 min.

In BMSC group (BG), the rats were given an intravenous (through caudal vein) injection of BMSC (2 × 10^6^ cells) at 10 min after seawater instillation as in SG, and the other treatment conditions were the same as those in SG.

In naive group (NG), neither seawater nor BMSC was given in this group. And the other treatment conditions were same as those in SG.

### 2.4. Arterial Blood Gas Analysis

The rats were sacrificed by aortic puncture at the indicated time points. The blood samples were immediately taken for arterial blood gas analysis. Hydrogen ion concentration (pH), arterial oxygen tension (PaO_2_), and arterial carbon dioxide tension (PaCO_2_) were measured with a blood gas analyzer.

### 2.5. Lung Weight Coefficient

The lung weight coefficient was determined as an index of pulmonary edema. The lung tissue were immediately removed and weighed after the surface blood was aspirated. The lung weight coefficient was calculated by dividing the lung weight by body weight in each rat.

### 2.6. Measurement of TNF-*α* and IL-8 in Bronchoalveolar Lavage Fluid (BALF)

The bronchoalveolar lavage fluid was taken by a method as described by Han et al. [16]. Briefly, the left lungs were excised integrally from rats in each group, the bronchoalveolar lavage (BAL) was made with intratracheal injections of 2 mL of physiological saline at 37°C three times. The BALF was retrieved and centrifuged, and TNF-*α* and IL-8 were then determined by the ELISA method (R&D Systems Inc., Minneapolis, MN, USA).

### 2.7. Histopathological Examination

Lung tissue for histological study was fixed in fresh 4% formaldehyde solution for 24 h and then dehydrated and embedded in paraffin, finally 4 *μ*m sections were cut and stained with hematoxylin-eosin (H&E). The tissue sections were observed under a light microscope for the lung histopathology. Lung injury was evaluated according to the degree of alveolar edema, interstitial edema, neutrophil infiltration, and hemorrhage.

### 2.8. RT-PCR

Total RNA was extracted from lung tissue using the Trizol reagent and reverse-transcribed into cDNA using commercial kits (Fermentas, Lithuania). PCR was carried out in 20 ul reaction volumes, containing 7 *μ*L of PCR Mix (10x Taq Buffer with (NH_4_)_2_SO_4_, 0.2 mmol/L dNTP, 1.5 mmol/L MgCl_2_), 1 *μ*L of each primer, 9 *μ*L ddH_2_O, and 2 *μ*L of genomic DNA. PCR cycling was performed using the MyCycler Thermal Cycler (Bio-Rad) with following conditions: at 95°C, 5 min, followed by 35 cycles of 30 sec at 95°C, 30 sec at 60°C, and 45 sec at 72°C, after cycles at 72°C, 10 min. The primer sequences and the expected sizes of PCR products were as follows. LC3 (sense): 5′-CCATGCCGTCCGAGAAGACCTTC-3′ and (antisense) 5′-GACCAGCTTCCGCTGGTAACGTC-3′ (452 bp). GAPDH: (sense) 5′-GCAAGTTCAACGGCACAG-3′ and (antisense) 5′-GCCAGTAGACTCCACGACAT-3′ (140 bp).

### 2.9. Western Blotting

In brief, lung tissue in each group was homogenized in ice-cold RIPA lysate buffer (Sigma) and centrifuged at 15000 ×g for 15 min at 4°C. The total protein concentration was determined with a UV 3000 ultraviolet spectrophotometer (Nano Drop, Wilmington, DE). The samples (100 ug protein per lane) were separated with 15% gradient SDS-PAGE gel and then transferred onto a PVDF membrane. Nonspecific binding to the membrane was blocked with 5% nonfat milk in Tris buffered saline-Tween 20 (TBST, pH 7.4) for 2 h at room temperature. After that the membranes were incubated overnight with rabbit polyclonal antibody against LC3 (1 : 1000 dilution, Cell signaling technology) and *β*-actin (1 : 1000 dilution, Sigma). After washed repeatedly with TBST, the membrane were incubated in goat anti-rabbit IgG (1 : 5000 dilution, Beijing Zhongshan Biotechnology Co., Ltd., Beijing, China) for 2 h, and bands were visualized by using the ECL kit (Proteintech, USA). The ratio of LC3 level II/LC3I level was used as an indicator of autophagic level.

### 2.10. Statistical Analysis

All statistical analyses were carried out using SPSS statistical software version 13.0 (SPSS, Chicago, USA). Data were summarized as mean ± SD; one-way ANOVA and post hoc comparisons were used to determine the difference among multiple groups. *P* < 0.05 was regarded as statistically significant.

## 3. Results

### 3.1. Effects of BMSC Treatment on Arterial Blood Gas

The previous study and our earlier experiments all found that the most obvious inflammatory response and autophagy of lung tissues occurred at about 2 h after seawater exposure [16]. Therefore the 2 h time point was considered suitable for the following studies.

**Figure 1:**
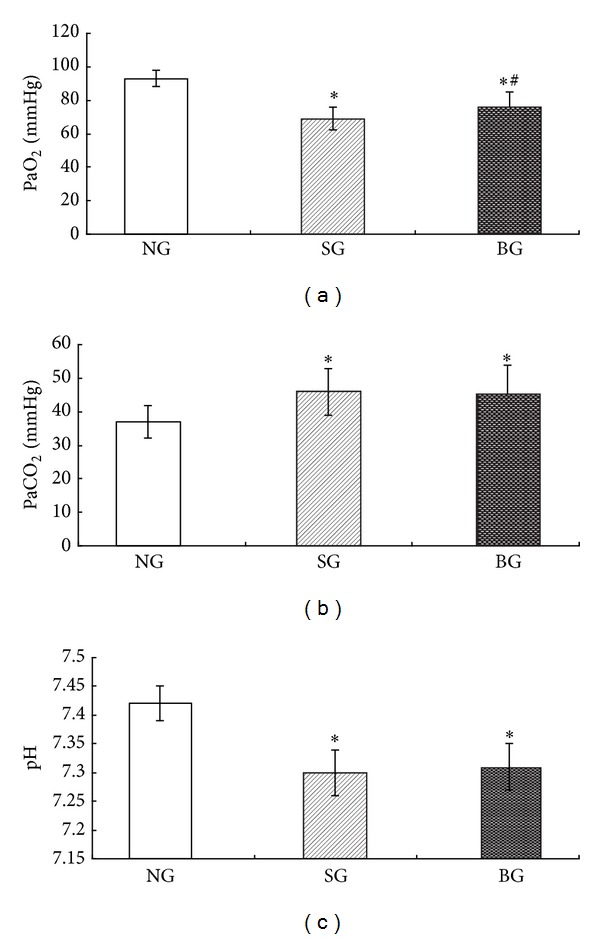
Effects of BMSC treatment on changes in arterial blood gas parameters at 2 h after seawater exposure. (a) PaO_2_; (b) PaCO_2_; (c) pH. Data are expressed as mean ± S.D. *P < 0.05 compared to NG group. ^#^ *P* < 0.05 compared to SG group.

Compared with the control group, seawater exposure caused significant changes in the arterial blood gas parameters, which was manifested by an obvious decrease in PaO_2_ and pH value, as well as a significant increase in PaCO_2_ (Figure 1).

As shown in Figure 1(a), the decrease of PaO_2_ induced by seawater instillation were significantly reversed by treatment of BMSC; however, the changes of PaCO_2_ and pH value were not significantly reversed (Figures 1(b) and 1(c)).

### 3.2. Effects of BMSC Treatment on Lung Weight Coefficient

**Figure 2:**
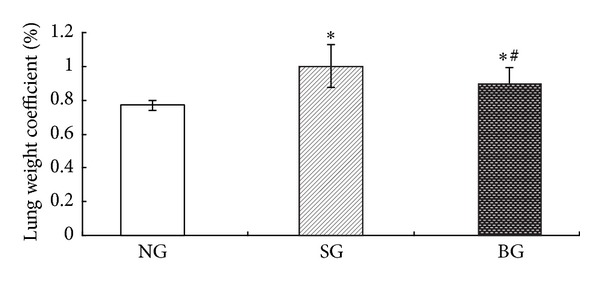
Effects of BMSC treatment on changes in lung weight coefficient at 2 h after seawater exposure. Data are expressed as mean ± S.D. *P < 0.05 compared to NG group. ^#^ *P* < 0.05 compared to SG group.

Lung weight coefficient was significantly increased in the seawater group when compared with that in the control group. Moreover, lung weight coefficient was partly ameliorated by treatment of BMSC (Figure 2).

### 3.3. Effects of BMSC Treatment on TNF-*α* and IL-8 in Bronchoalveolar Lavage Fluid (BALF)

As shown in Table 1, TNF-*α* and IL-8 levels in bronchoalveolar lavage fluid (BALF) significantly increased in the SG when compared with those in the NG. In contrast, IL-8 level in bronchoalveolar lavage fluid (BALF) was slightly attenuated by treatment of BMSC when compared with SG (Table 1). However, TNF-*α* level in bronchoalveolar lavage fluid (BALF) was not obviously ameliorated by treatment of BMSC (Table 1).

### 3.4. Effects of BMSC Treatment on Lung Histopathological Changes

**Figure 3:**
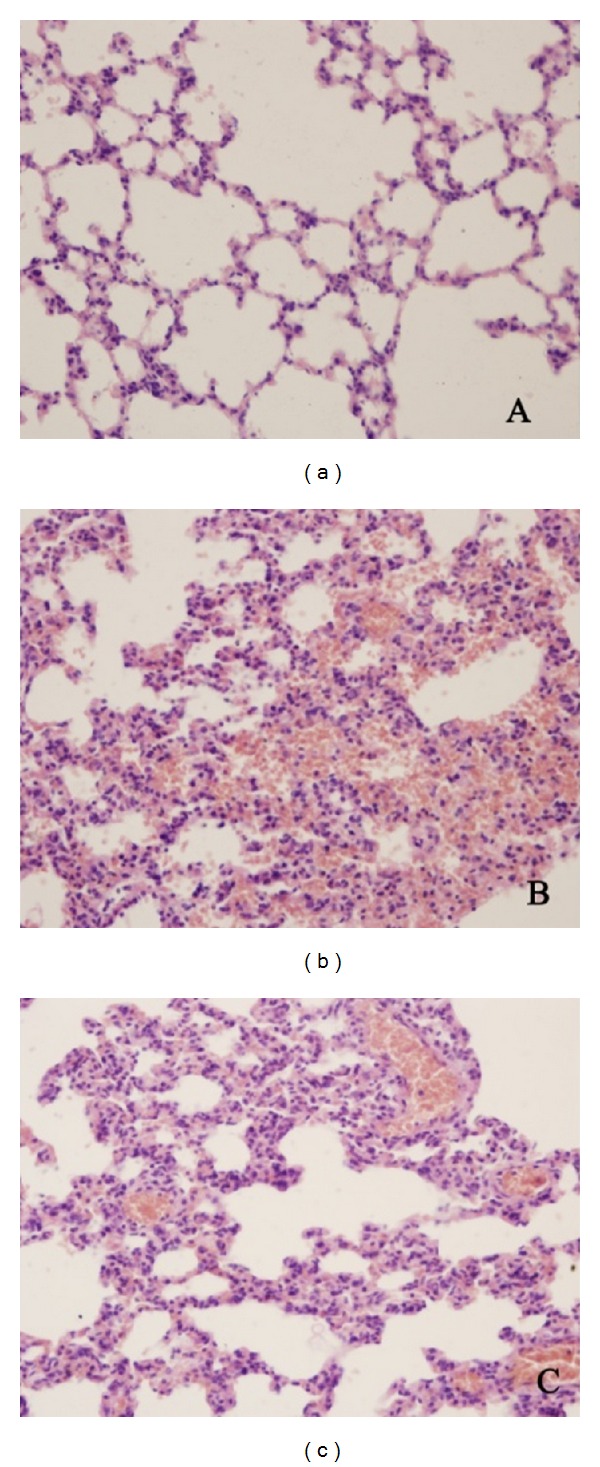
Light micrographs of lung tissues stained with haematoxylin-eosin (H&E) (×400). (a) Lung tissue in a rat of NG group. (b) Lung tissue in a rat of SG group. Hemorrhage, edematous changes, collapse, and inflammatory exudation in alveolar were observed. (c) Lung tissue in a rat of BG group. Lung injury was alleviated and less damage was observed compared with the SG group.

As H&E (haematoxylin and eosin) stained sections showed, normal lung tissue structures and clear alveoli existed in rats of NG group (Figure 3(a)). Seawater exposure induced prominent lesions, such as edema and hemorrhage, collapse, and inflammatory exudation in alveolar (Figure 3(b)). In contrast, these changes were partly ameliorated by BMSC treatment.

### 3.5. Effects of BMSC Treatment on LC3 mRNA Level by RT-PCR

**Figure 4:**
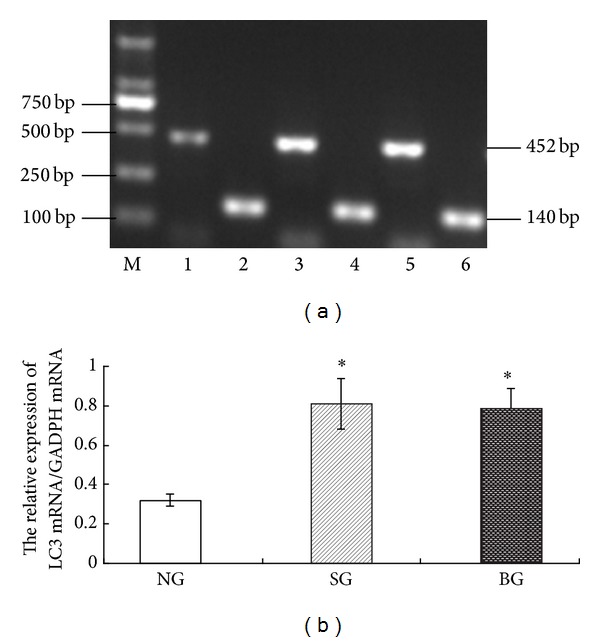
Effects of BMSC treatment on LC3 mRNA level in rat lung tissue. (a) Electrophorogram picture for LC3 mRNA in lung tissues in different groups. (b) The ratio of LC3 mRNA/GADPH mRNA was presented in bar chart. *P < 0.05, compared with NG group. M: marker; lanes 1, 3, and 5 represent the LC3 mRNA expression (452 bp) in NG, SG, and BG groups, respectively; lanes 2, 4, and 6 represent the GADPH mRNA expression (140 bp) in NG, SG, and BG groups, respectively.

Autophagy-related (Atg) proteins are eukaryotic factors participating in various stages of the autophagic process. Thus far 34 autophagy-related genes (Atg) have been identified in yeast [17]. Recently, the orthologs of Atg genes have been isolated and functionally characterized in mammals [18]. LC3 is a mammalian homologues protein of yeast Atg8 protein; LC3 gene is one of critical genes involved in autophagy [19]. Therefore, we focused on the changes of LC3 gene in this study. RT-PCR results showed that there was a notable upregulation of LC3 mRNA level in rat lung tissue of SG group when compared with NG group (Figure 4). Furthermore, the activation of LC3 after seawater exposure was slightly reversed by BMSC treatment.

### 3.6. Effects of BMSC Treatment on LC3 Conversion by Western Blotting

**Figure 5:**
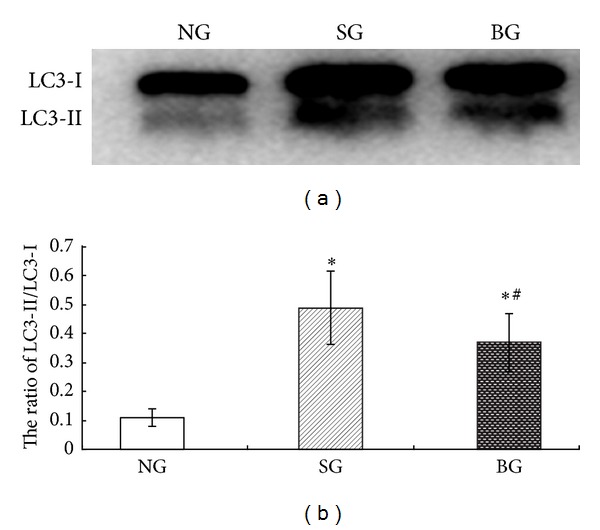
Effects of BMSC treatment on LC3 conversion in rat lung tissue. (a) Western blot picture for LC3 in lung tissues in different groups. (b) The ratio of LC3-II/LC3-I was presented in bar chart. *P < 0.05, compared with NG group. ^#^ *P* < 0.05 compared to SG group.

The conversion of LC3-I to LC3-II is a necessary step during the process of autophagy. The level of LC3-II is known to correlate well with the number of autophagosomes. Our studies showed that the LC3-II protein was hardly detectable in normal lung tissue by using western blotting analysis. However, there was a marked upregulation of the ratio of LC3-II/LC3-I in rats of SG group (Figure 5). What is more, these changes were partly reversed by BMSC treatment.

## 4. Discussion

In the present study, we investigated the role of BMSC transplantation for early seawater-induced ALI in a rat model. The results demonstrated that seawater exposure induced ALI including impaired arterial blood gas, pulmonary edema, histopathologic changes, and inflammatory response in lung tissues. What is more, these changes were partly ameliorated by BMSC treatment through inhibiting autophagy in lung tissues.

Drowning is one of the most common causes of accidental deaths in the world [16]. Actually, there are mostly two kinds of outcomes after drowning. Some victims might die of apnoea soon after drowning; other patients might survive initial apnoea and then suffer acute lung injury [20]. The aim of this study was to explore the therapeutic effect of BMSC transplantation in early seawater-induced ALI, so we only chose the rats that survived the early stage after seawater intratracheal instillation for further experiments. Furthermore, previous studies and our earlier experiments showed that the most obvious inflammatory response and autophagy of lung tissues occurred at about 2 h after seawater exposure [15, 16]. Therefore the 2 h time point was selected for the following studies.

In this study, we found that seawater exposure could cause lung injury, which was characterized by decreased PaO_2_ and increased PaCO_2_, lung weight coefficient, and TNF-*α* and IL-8 in bronchoalveolar lavage fluid (BALF) in a rat model. The results were consistent with histopathologic findings. All these data suggested that the intratracheal exposure of seawater in the rat model induced topical acute lung injuries (ALI), which was also in accordance with the findings of other authors and our previous study [2, 15, 16, 21].

In addition, we explored whether or not the BMSC treatment played a role in early seawater-induced ALI in a rat model. We found that PaO_2_, lung weight coefficient, histopathologic changes, and inflammatory responses induced by seawater exposure were partly attenuated by treatment of BMSC. It indicated that BMSC might play a protective effect on the ALI induced by seawater exposure. Bone mesenchymal stem cells (BMSC) were originally identified more than 120 years ago as a component population among the predominant hematopoietic cells of the bone marrow [22]. Furthermore, BMSC express no major histocompatibility complex class II; they are the viable therapeutic methods across tissue typing [23]. It also has been shown to differentiate into a variety of mesenchymal cell types, including fibroblasts, myofibroblasts, osteoblasts, chondroblasts, adipocytes, and myoblasts, as well as epithelial cells [5]. In addition, BMSC might provide a number of potent cytokines and growth factors to protect lung injury [5]. Our current finding is in agreement with other studies which have shown an ameliorating effect of BMSC treatment in other models of ALI [6–9]. Xu et al. found that intravenous transplantation of BMSCs could maintain the integrity of the pulmonary alveolar-capillary barrier and modulate the inflammatory response to attenuate oleic-acid-inducing the experimental ALI, and transplantation of BMSCs could be a novel cell-based therapeutic strategy for prevention and treatment of ALI [6]. Huang et al. reported that BMSCs had a protective effect on paraquat-induced ALI in rats, and the effect was closely related to the transplantation time and number of transplanted BMSCs [7]. The study of Curley et al. showed that BMSC therapy enhanced lung repair following ventilator-induced ALI via a paracrine mechanism that might be dependent on keratinocyte growth factor [8]. Islam et al. argued that BMSC protected against ALI by restituting alveolar bioenergetics through Cx43-dependent alveolar attachment and mitochondrial transfer [9].

Furthermore, our present study, reported that ALI induced by seawater exposure was ameliorated by BMSC through inhibiting autophagy of lung tissues in a rat model. Autophagy, a process in which de novo formed membrane-enclosed vesicles engulf and consume cellular components, has been shown to induce type II programmed cell death when it is inappropriately activated [24]. Evidence of autophagy in the pathogenesis of ALI has gradually accumulated in recent years [14]. For example, Sun et al. concluded that the autophagic cell death of alveolar epithelial cells likely plays a crucial role in the high mortality rate of H5N1 infection and autophagy-blocking agents might be useful as prophylactics and therapeutics against infection of humans by the H5N1 virus [13]. Li et al. reported that nanoparticles triggers autophagic cell death and the autophagy inhibitor 3-methyladenine might rescue cell death and ameliorate ALI caused by nanoparticles in mice [12]. Our data showed that LC3 II, an indicator of autophagosome formation, was attenuated by BMSC treatment. The alterations of autophagy were basically consistent with the changes in arterial blood gas, lung weight coefficient, TNF-*α* and IL-8 in bronchoalveolar lavage fluid (BALF), and histopathologic changes. These results demonstrated that autophagy was involved in the ALI pathophysiological process. Moreover, this is the first evidence, to our knowledge, that BMSC treatment protects against seawater-induced ALI by inhibition of autophagy in lung tissue. Taken together, our study provides evidence to further support the view that autophagy-blocking might be useful therapy for ALI.

In summary, data in this study demonstrated that BMSC treatment may exert some protective effects on ALI induced by seawater exposure, partly through inhibiting autophagy in lung tissue. The application of BMSC may be a potential effective treatment for ALI. However, further investigations on the effect of BMSC in long time are still needed in the future.

## Figures and Tables

**Table 1:** Effects of BMSC treatment on changes in TNF-*α* and IL-8 in bronchoalveolar lavage fluid after seawater exposure in rats.

Group	TNF-*α* (pg/mL)	IL-8 (pg/mL)
NG	21.67 ± 6.86	30.61 ± 5.28
SG	52.83 ± 16.74^∗^	55.50 ± 8.96^∗^
BG	52.17 ± 10.36^∗^	52.01 ± 11.31^∗^

^∗^ *P* < 0.05, compared with NG.
